# Positive end-expiratory pressure titration in acute respiratory distress syndrome–a practical bedside algorithm

**DOI:** 10.3389/fmed.2026.1807478

**Published:** 2026-06-24

**Authors:** Luís Melo, João João Mendes

**Affiliations:** 1 Serviço de Medicina Intensiva, Unidade Local de Saúde de Santa Maria, Lisbon, Portugal; 2 Clínica de Medicina Intensiva da Faculdade de Medicina da Universidade de Lisboa, Lisbon, Portugal; 3 Serviço de Medicina Intensiva, Unidade Local de Saúde Amadora-Sintra, Lisbon, Portugal; 4 Unidade de Cuidados Intensivos Polivalente, Hospital CUF Tejo, Lisbon, Portugal

**Keywords:** ARDS, mechanical ventilation, physiological monitoring, positive end-expiratory pressure, recruitment maneuvers

## Abstract

Positive end-expiratory pressure (PEEP) selection and titration is a longstanding debate in management of mechanically ventilated patients with acute respiratory distress syndrome (ARDS). Although multiple strategies have been tested, heterogeneity in ARDS physiology, co-interventions, and variable recruitability have limited clear conclusions. Advanced monitoring may support individualized titration, but availability and outcome evidence remain limited. In this article we present a narrative review of the evidence behind different PEEP setting strategies and propose a practical bedside algorithm in 5 steps to individualize PEEP settings. It incorporates guideline-protective ventilation, assessment of recruitability, prone position and consideration of rescue therapies. The proposed algorithm is guided by physiology and readily available ventilator-derived parameters, intended to be used by any clinician at the bedside.

## Introduction

The precise titration of PEEP remains a fundamental component in the management of acute hypoxemic respiratory failure. Despite advances in the understanding of ARDS pathophysiology, determining the optimal balance between enhancing oxygenation, maintaining alveolar stability, preventing overdistension, and ensuring systemic oxygen delivery continues to pose considerable clinical challenges. Although advanced monitoring techniques are becoming more widely employed, and show promising improvement in physiological parameters, they are still unavailable in many clinical settings.

This narrative review was informed by the authors’ clinical experience, which guided prioritization of foundational papers, influential recent studies, and areas of ongoing debate. It synthesizes current evidence-based approaches, with an emphasis on widely accessible bedside tools, and discusses how PEEP adjustment affects recruitment, overdistension, gas exchange, and hemodynamic tolerance.

## Pathophysiology of ARDS and the functional role of PEEP

ARDS is characterized by heterogeneous alveolar collapse, gravity-dependent atelectasis, and intrapulmonary shunting, all of which contribute to severe ventilation–perfusion (V/Q) mismatch, hypoxemia, and impaired gas exchange (1). Inflammatory pulmonary edema increases total lung weight, and in the supine position, this additional mass promotes dorsal lung collapse, displacing ventilation toward non-dependent regions – a phenomenon known as compression atelectasis. Despite their collapse, dorsal regions often remain perfused, perpetuating right-to-left shunt and worsening oxygenation (2).

The “baby lung” concept, first proposed by Gattinoni et al. (3), describes the markedly reduced volume of normally aerated lung tissue in severe ARDS – approximately 200-500 grams at end-expiration on computed tomography, equivalent to the lung size of a healthy 5-year-old child. The proportion of nonaerated tissue correlates with the severity of hypoxemia, shunt fraction, and pulmonary hypertension, whereas respiratory system compliance reflects the volume of normally aerated lung (4). Thus, the ARDS lung is not uniformly stiff but rather functionally small, with the remaining ventilated regions often exhibiting near-normal elasticity.

In the supine position during mechanical ventilation, most tidal volume is delivered to the ventral, more compliant, non-dependent regions, increasing local stress and strain. Conversely, dorsal lung areas – more prone to collapse – receive less ventilation and may undergo cyclic recruitment and derecruitment, generating shear stress and amplifying lung injury. The distribution of alveolar opening pressures in ARDS is typically unimodal, peaking between 20 and 25 cmH₂O, although some regions require pressures as high as 45 cmH₂O for reopening (5). Consequently, during tidal inspiration, low-threshold alveoli recruit first, while high-threshold units remain collapsed if plateau pressures are limited, leading to persistent atelectasis and overdistension of ventilated areas. Without adequate PEEP, newly recruited units at peak inspiration frequently derecruit at end-expiration, causing surfactant depletion and further alveolar injury.

Appropriately set PEEP counteracts these compressive forces; promotes more homogeneous ventilation, and lowers the risk of ventilator-induced lung injury (VILI) – a central therapeutic goal in ARDS (6). In 1975, Suter et al. proposed the concept of “optimal PEEP,” defined as the level of PEEP that results in the highest static respiratory compliance. This is the level that yields maximal alveolar recruitment, minimal adverse hemodynamic effects, and optimal oxygen delivery (DO_2_). This seminal observation highlighted the need to integrate respiratory mechanics, hemodynamics, and gas exchange parameters when titrating PEEP (7).

## Airway closure

Airway closure is an important, yet frequently under-recognized, phenomenon in mechanical ventilation – particularly in ARDS and other forms of acute respiratory failure. It refers to the cessation of airflow due to the collapse of small conducting airways at low lung volumes, resulting in pronounced V/Q mismatch.

In healthy, spontaneously breathing individuals, small airway closure is a physiological event of limited magnitude, typically occurring in dependent lung regions toward the end of expiration (8).

In ARDS, airway closure is amplified by altered surface tension, airway liquid redistribution, and mechanical interdependence between alveolar units (9). As expiration progresses, closure begins in dependent regions and may propagate to other lung zones. Cyclic closure and reopening can generate shear stress and contribute to regional inflammation (10, 11).

**Figure 1:**
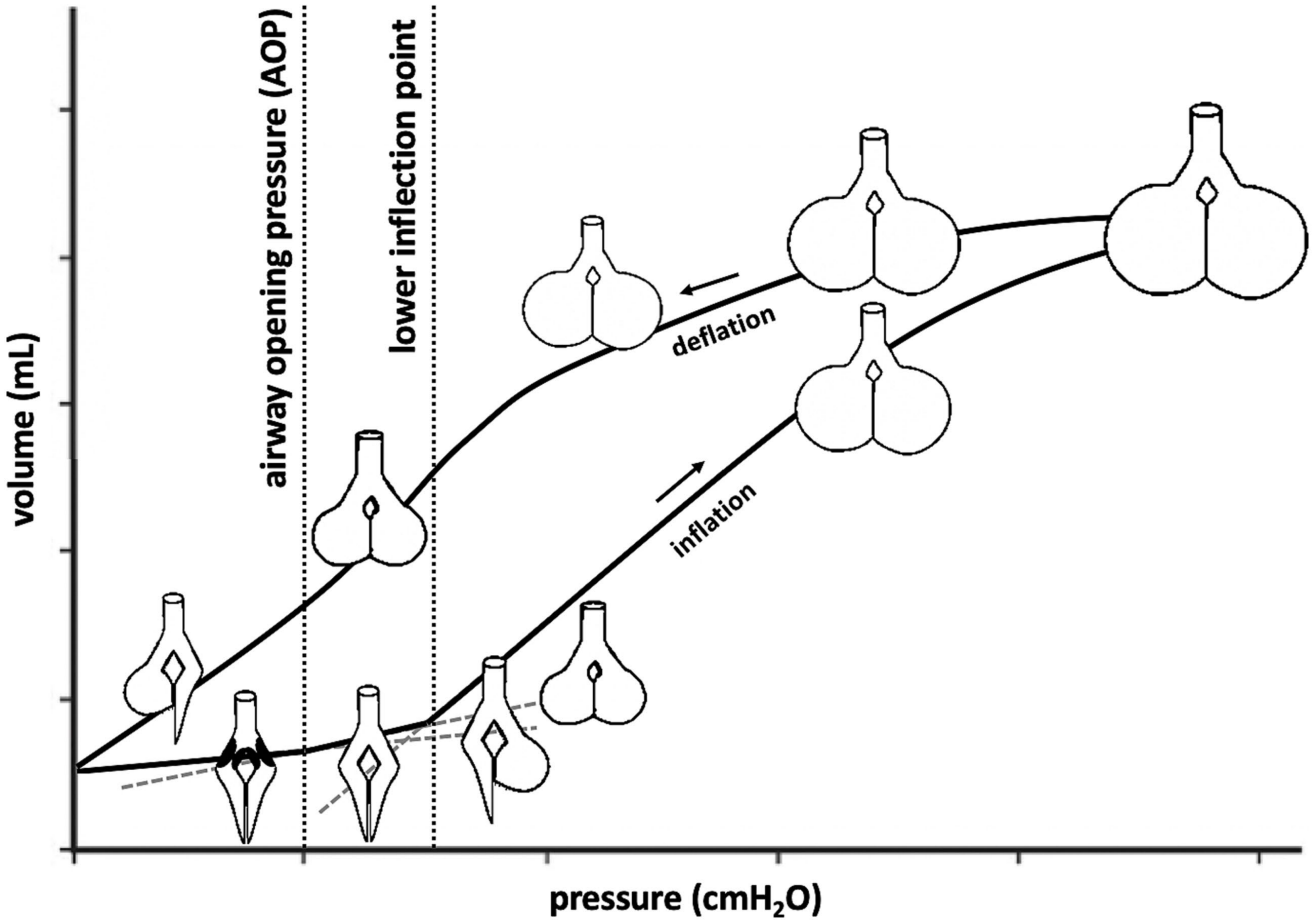
Pressure–volume curve with hysteresis. Schematic pressure-volume relationship showing airway opening pressure (AOP) and lower inflection point. The hysteresis area of the pressure–volume curve is the area enclosed between the inspiratory and expiratory limbs, reflecting alveolar recruitment dynamics during inflation and deflation and representing dissipated mechanical energy within the respiratory system.

Lung inflation begins only once airway pressure exceeds a critical airway opening pressure (AOP) (Figure 1). Up to this point, pressure generated by the ventilator is not transmitted to the alveoli and produces negligible changes in lung volume. Historically, this inflection has sometimes been misinterpreted as the “lower inflection point” on the pressure-volume curve, assumed to indicate alveolar recruitment. While airway closure and atelectasis frequently coexist, they are distinct processes: atelectasis refers to alveolar collapse, whereas airway closure denotes obstruction of small conducting airways (12). Notably, airway closure can be present in both atelectatic lungs (as in ARDS) and hyperinflated lungs (as in asthma). In the latter scenario, some alveoli may remain inflated but disconnected from proximal airways. Finally, airway closure compromises the accuracy of bedside respiratory mechanics measurements. Driving pressure and compliance calculated from airway opening may be misleading if distal airway obstruction prevents full pressure equilibration between the ventilator and alveolar spaces (13).

**Figure 2:**
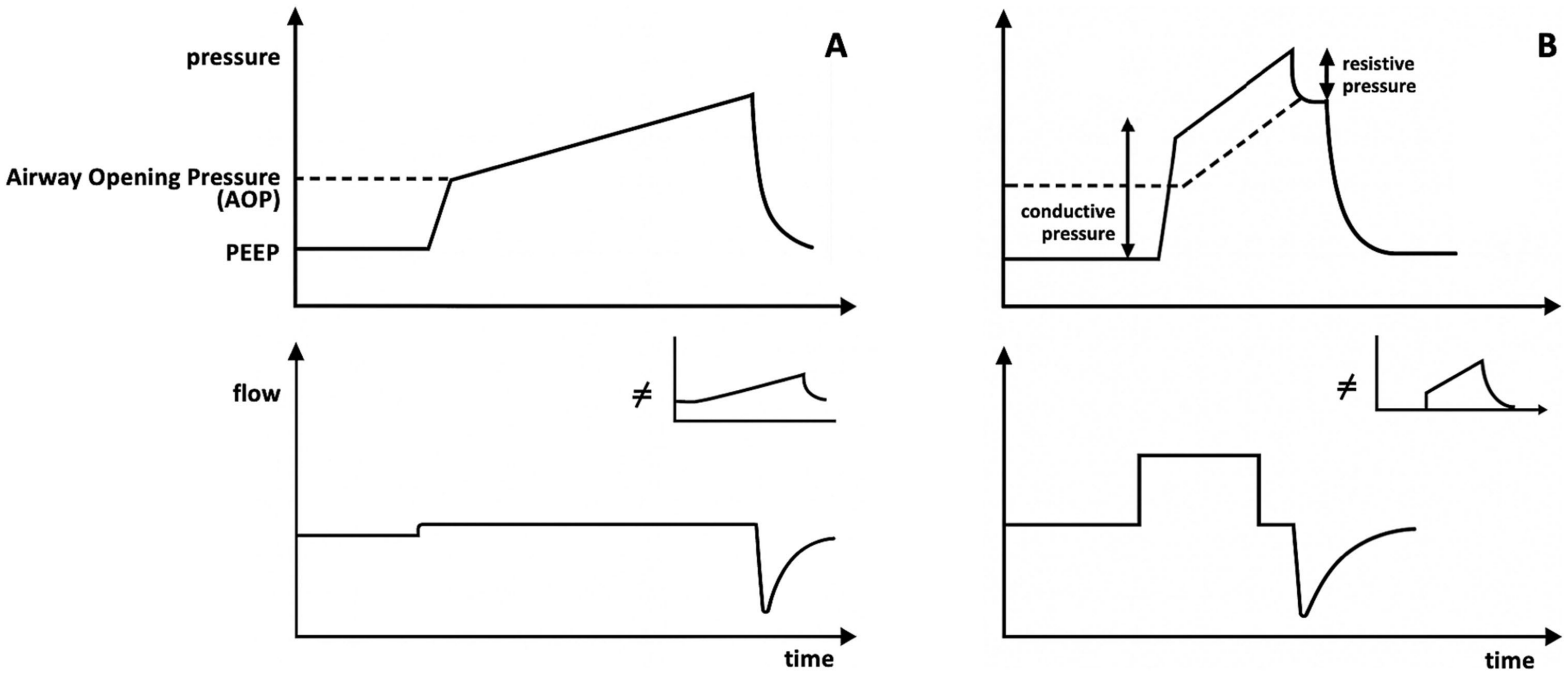
Bedside assessment of airway opening pressure. **(A)** Low-flow insufflation method: during a slow inflation, a sudden volume increase at minimal pressure rise indicates AOP; **(B)** Conductive pressure method: in constant-flow ventilation, a biphasic pressure–time curve with an initial steep rise followed by a slope change identifies AOP, with conductive pressure exceeding normal resistive pressure.

AOP can be estimated at the bedside using low-flow insufflation or conductive pressure:

- Low-flow insufflation maneuver (Figure 2A): During a slow inflation, a sudden increase in delivered volume for minimal pressure rise marks the AOP, reflecting the point at which previously closed small airways reopen (9, 12, 13).
- Conductive pressure method (Figure 2B): Evaluates the early pressure–time curve during constant-flow volume-controlled ventilation. A biphasic pattern with an initial steep rise followed by a slope change suggests airway closure, with conductive pressure exceeding normal resistive pressure by >1 cmH₂O indicating the presence of AOP (14).

## Benefits and risks of PEEP

The application of PEEP was first proposed by Ashbaugh et al. as a means to counteract alveolar collapse driven by surfactant dysfunction in ARDS (15). Subsequent studies confirmed that PEEP primarily improves arterial oxygenation by recruiting collapsed lung units, thereby enhancing ventilation–perfusion matching and reducing intrapulmonary shunt (16). Animal models have consistently demonstrated that PEEP mitigates ventilator-induced lung injury (VILI) by reducing atelectrauma (17, 18), increasing functional residual capacity and decreasing tidal stress and strain (19–21).

However, if PEEP overdistends already aerated regions or diverts perfusion away from ventilated lung, it can increase V/Q mismatch. Physiologic data show that improved aeration with higher PEEP does not always translate into better V/Q matching (22), and an elevated dead-space fraction is independently associated with worse outcomes in ARDS (23). Changes in lung volume induced by PEEP also influence pulmonary vascular resistance, with direct implications for right ventricular function. Collapsed alveoli exhibit elevated vasomotor tone due to hypoxic pulmonary vasoconstriction. PEEP can reverse these effects, thereby reducing pulmonary vascular resistance (15, 24). However, excessive PEEP may also increase transpulmonary pressure, reducing alveolar capillary cross-sectional area, and ultimately elevating pulmonary vascular resistance. The relationship between pulmonary vascular resistance and lung volume follows a U-shaped curve, with the nadir at functional residual capacity (25).

Beyond pulmonary effects, PEEP increases intrathoracic and pleural pressures, consequently raising right atrial pressure and reducing the gradient for venous return. This decrease in preload may reduce cardiac output. Paradoxically, the resulting decline in cardiac output may improve V/Q matching by redistributing pulmonary blood flow, thereby increasing arterial oxygen tension (PaO₂) despite reduced systemic oxygen delivery (26, 27). Thus, PEEP selection based solely on PaO₂/FiO₂ targets – without considering its hemodynamic consequences – may lead to suboptimal outcomes (28).

The hemodynamic impact of excessive PEEP is often immediately evident at the bedside, whereas its effects on lung mechanics may be less readily obvious. In cases where PEEP does not lead to a substantial recruitment of lung volume, both dynamic and end-inspiratory stress may increase, promoting overdistension and triggering inflammatory cascades akin to those induced by excessive tidal volumes (29). More immediately obvious complications of excessive PEEP include barotrauma, with potential development of pneumothorax or subcutaneous emphysema. These risks highlight the need for integrated monitoring of respiratory mechanics, gas exchange, and hemodynamics when titrating PEEP.

### Clinical evidence supporting PEEP titration

The ARDS Network ARMA trial (30) was the pivotal randomized controlled study establishing lung-protective ventilation as the standard of care in ARDS. The protocol applied a PEEP/FiO₂ table, assigning specific PEEP levels according to the inspired oxygen fraction required to maintain target oxygenation (PaO₂ 55–80 mmHg or SpO₂ 88–95%). This approach aimed to avoid excessive PEEP while strictly limiting plateau pressure to ≤ 30 cmH₂O, thereby minimizing volutrauma and barotrauma. These tables have since been used extensively in several randomized controlled trials, offering a simple, reproducible framework for PEEP settings. However, they do not account for patient-specific respiratory mechanics or lung recruitability, which may lead to suboptimal ventilation in cases with heterogeneous disease patterns.

Four large clinical trials – ALVEOLI, LOVS, EXPRESS, and ART (24, 31, 31–) – have compared different PEEP-setting strategies, with or without recruitment maneuvers. Three of these trials found improved oxygenation and reduced need for rescue therapies without a mortality benefit, whereas the ART trial reported increased 28-day mortality and higher rates of barotrauma and hemodynamic instability. The aggressive recruitment strategy in ART – stepwise PEEP titration to 45 cmH₂O and plateau pressures up to 60 cmH₂O – may have contributed to these adverse outcomes. Furthermore, in ART, the “low PEEP” group had a mean PEEP of 12 cmH₂O, which was higher than in the control arms of other trials.

Notably, none of these trials incorporated pre-intervention assessment of lung recruitability, potentially exposing low-recruitability patients to unnecessarily high airway pressures. This factor may have masked benefits in patients with high recruitability while increasing harm in those with low recruitability. A meta-analysis including ALVEOLI, LOVS, and EXPRESS suggested a potential benefit of higher PEEP in patients with PaO₂/FiO₂ ≤ 200 (32), supporting the hypothesis that baseline severity and recruitability influence PEEP responsiveness.

Overall, these findings highlight the limitations of a one-size-fits-all approach in a syndrome as heterogeneous as ARDS. Individualized PEEP titration strategies – guided by physiologic assessment of recruitability – may optimize outcomes while minimizing risk.

### Driving pressure and PEEP

Driving pressure (ΔP), defined as plateau pressure minus PEEP, reflects the pressure required to deliver tidal volume to the ventilated “baby lung.” At a fixed tidal volume, changes in driving pressure after PEEP adjustment therefore reflect changes in respiratory system compliance and may help distinguish recruitment from overdistension. For this reason, ΔP is a practical bedside surrogate of cyclic lung strain and is likely more relevant than tidal volume, plateau pressure, or PEEP considered in isolation. This framework was established most clearly by Amato and colleagues, who showed that much of the apparent benefit of low-VT and higher-PEEP strategies could be explained through reductions in driving pressure, reinforcing the idea that the physiologic effect of a PEEP intervention matters more than the absolute level itself (33).

The relationship between ΔP and PEEP is nonlinear and patient-specific. At a fixed tidal volume, any change in driving pressure after a PEEP adjustment reflects a change in respiratory system compliance. If a PEEP increase prevents end-expiratory collapse, compliance improves and plateau pressure rises less than the increment in PEEP, causing ΔP to fall. In contrast, if the same PEEP increase primarily over-distends already open lung units, compliance worsens, plateau pressure rises disproportionately, and ΔP increases (33, 34).

Conceptually, the lowest driving pressure is expected near the high-compliance portion of the pressure-volume curve, where alveoli remain open at end-expiration without substantial over-inflation during inspiration. Physiologic studies also demonstrated that driving pressure equilibrates within approximately 1–5 min after a PEEP change (35, 36). This suggests that lung recruitment is also time-dependent and that evaluation of lung distending pressures should be performed after a period of stabilization.

Accordingly, a bedside PEEP titration algorithm should not pursue high PEEP per se, nor treat oxygenation alone as proof of benefit. Instead, it should identify the PEEP at which driving pressure is minimized, while plateau pressure, gas exchange, and hemodynamics remain acceptable.

### Recruitment maneuvers in ARDS

Each alveolus requires a minimum pressure to overcome surface tension forces and reopen. In ARDS, this alveolar opening pressure varies considerably across lung regions (21). Conventional protective ventilation often leaves portions of the potentially recruitable lung collapsed. In early ARDS, computed tomography (CT) data from Cressoni et al. (37) demonstrated that even at 30 cmH₂O airway pressure, 10-30% of recruitable lung tissue remained non-aerated in patients with moderate to severe disease (38). Recruitment maneuvers are defined as a temporary increase in airway and transpulmonary pressure, to values higher than encountered during tidal ventilation, for the goal of promoting re-aeration of previously gasless regions and restore lung volume closer to functional residual capacity. Once recruitment is achieved, adequate PEEP must be applied to prevent recurrent collapse. Definitions of “recruitment” vary according to how it is assessed: CT-based techniques, often referred to as the gold-standard, quantify recruitment as the volume or mass of previously non-aerated lung regaining aeration (reflected by decreased radiodensity) (39), whereas respiratory mechanics-based methods infer recruitment from compliance changes (40). These changes incorporate both newly opened alveoli and improved distensibility of previously aerated regions (41). These differing definitions will often yield divergent assessments of lung recruitability, and therefore the concept remains elusive.

**Figure 3:**
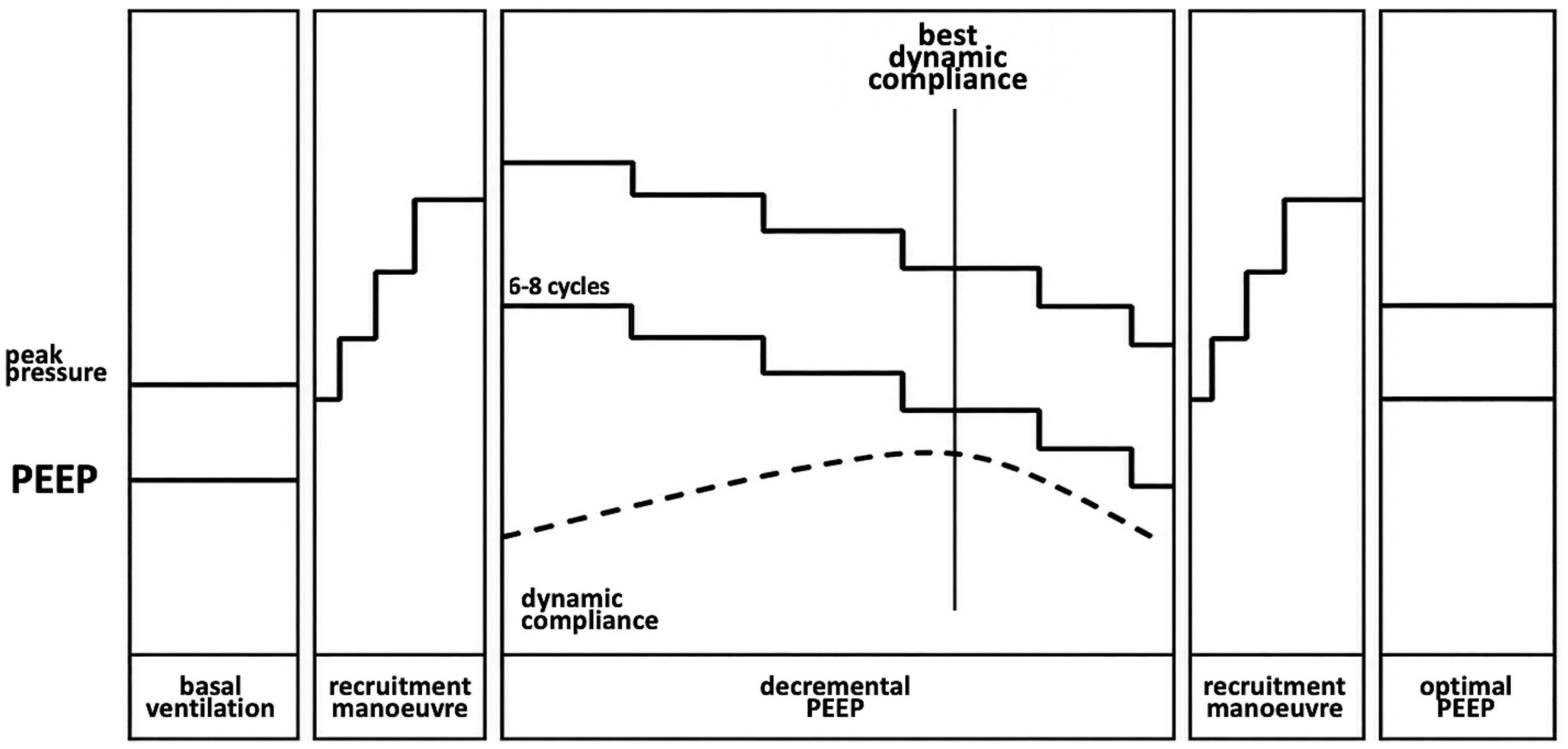
Stepwise recruitment maneuver and decremental PEEP trial. Illustration of a possible recruitment process followed by a decremental PEEP trial. Using pressure-controlled ventilation, increase PEEP in 5 cmH₂O increments every 5 s up to 30 cmH₂O, with inspiratory pressure set 15 cmH₂O above PEEP. After the recruitment maneuver, PEEP is decreased stepwise over 6-8 cycles while monitoring dynamic compliance. The optimal PEEP is set 2 cmH₂O above the level corresponding to best dynamic compliance, balancing alveolar recruitment and overdistension.

They can be divided in two main groups:

- Stepwise Recruitment Strategy – Gradual PEEP increments in small steps, followed by a decremental PEEP trial to identify the optimal pressure that maintains recruitment (Figure 3). This method aims to minimize hemodynamic compromise and barotrauma. In experimental acute lung injury, Silva et al. (42) showed that stepwise increases reduced static elastance and improved respiratory mechanics with less biological injury compared to sustained continuous positive airway pressure maneuvers. Kung et al. (43) demonstrated in early ARDS patients that stepwise recruitment maneuvers improved compliance, reduced extravascular lung water, and increased ventilator- and ICU-free days among survivors.
- Maximal Recruitment Strategy – Utilizes high PEEP and inspiratory pressures in a “one-step” increase that is sustained for at least 1 min, to achieve near-complete lung recruitment, followed by down-titration to maintain the recruited state. Five randomized clinical trials have analyzed the applications of maximal recruitment maneuvers (43–47). While no benefits in patient-centered outcomes were identified, two trials (PHARLAP trial (45) and ART trial (24)) reported a greater risk of barotrauma and hemodynamic instability.

Given these results, guidelines from the American Thoracic Society (ATS) and European Society of Intensive Care Medicine (ESICM) recommend against routine recruitment maneuvers, particularly in the case of maximal recruitment strategies, given the absence of clear mortality benefit and the signal for potential risks (48, 49). However, a recent systematic review and meta-analysis by Goligher et al. (50) found that recruitment maneuvers, particularly when combined with higher PEEP, were associated with reduced mortality and significant oxygenation improvement, without increased barotrauma or hemodynamic compromise. A Cochrane review by Hodgson et al. (51) indicates that protocol heterogeneity, frequent co-interventions (e.g., higher PEEP), and absence of testing for recruitability likely contribute as confounding factors regarding the potential benefits.

Recruitment duration also remains a topic of discussion and investigation. In a prospective study of 55 ARDS patients, Arnal et al. (52) found that 98% of recruited volume during a sustained inflation at 40 cmH₂O occurred within 10 s, whereas hemodynamic compromise typically began after this timepoint – supporting the concept that shorter durations of high pressures may be equally effective but safer.

In summary, recruitment maneuvers can improve oxygenation and may confer survival benefit in selected ARDS patients. However, they have been associated with increased risk of complications, namely hypotension and barotrauma and are not recommended for routine use in both ATS and ESICM guidelines. Their implementation requires individualized assessment of benefit, careful hemodynamic monitoring, and integration with PEEP optimization strategies to balance potential benefits against the risks of overdistension, barotrauma and cardiovascular instability.

### Evaluation of lung recruitability

Until recently, clinicians lacked practical, reliable bedside tools to assess lung recruitability in acute respiratory failure. Although repeated computed tomography (CT) scanning remains the reference standard, its clinical utility is limited by cost, radiation exposure, and the need for patient transport – factors that confine its use largely to research settings. An alternative, the multiple pressure-volume (P-V) curve method, exploits the hysteresis of the respiratory system to estimate recruitment (53). However, this approach is technically demanding, requiring construction and alignment of P-V curves from different starting lung volumes, and is not widely adopted in routine care (40).

Randomized controlled trials comparing recruitment maneuvers and PEEP strategies have rarely stratified patients by lung recruitability (24, 43, 45, 46, 50, 51). This omission likely contributes to heterogeneity in outcomes, as treatment effects may differ substantially between high- and low-recruitability phenotypes. A reproducible, simple, and real-time bedside method to quantify recruitability could therefore enhance individualization of ventilatory management.

**Figure 4:**
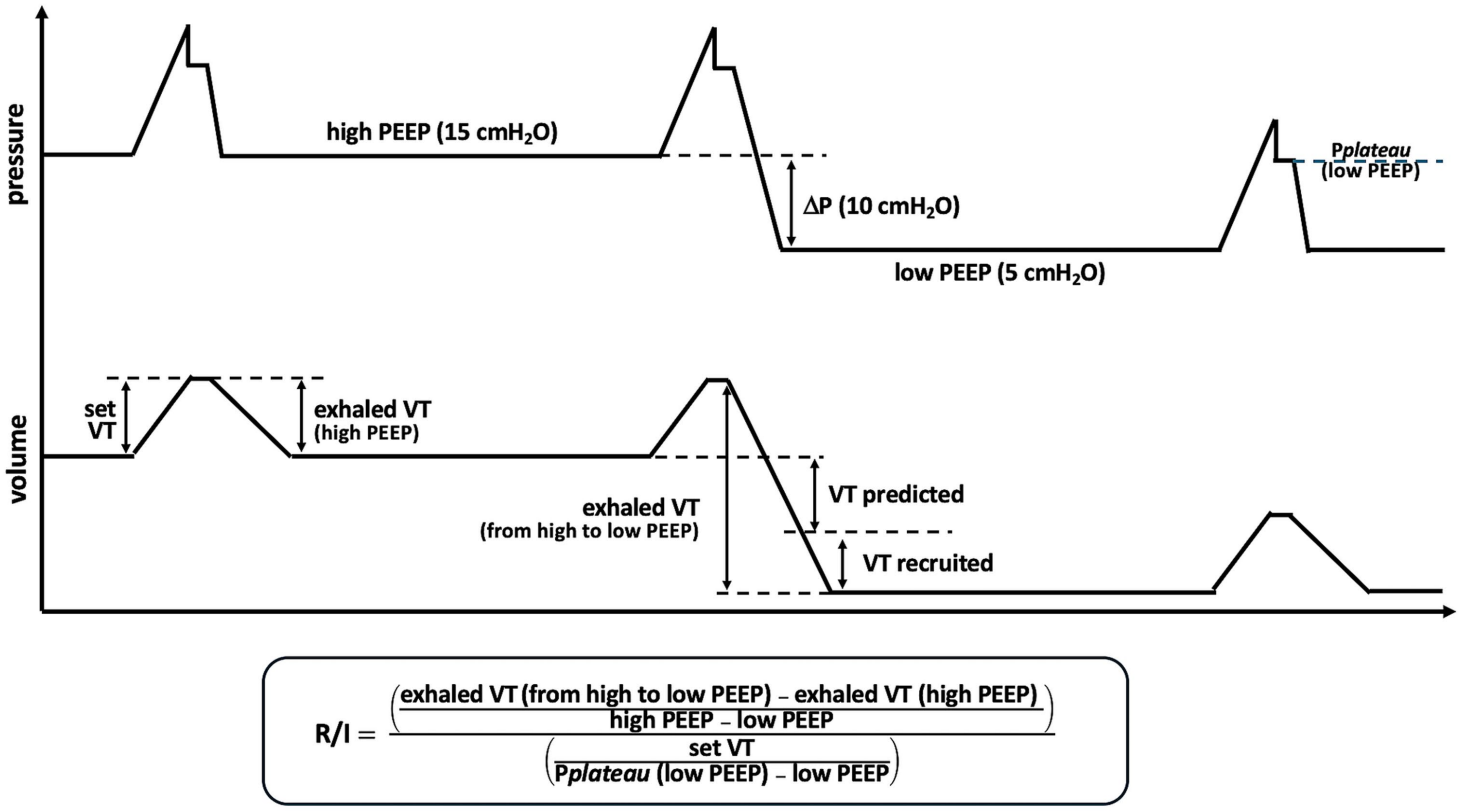
Single-breath decremental PEEP maneuver for Recruitment-to-Inflation ratio. Illustration of the method to assess lung recruitability at the bedside. The maneuver consists of: (1) reducing respiratory rate to exclude auto-PEEP; (2) measuring expired tidal volume at baseline PEEP (pre-reduction); (3) acutely lowering PEEP by 10 cmH₂O; (4) measuring expired tidal volume after PEEP reduction; and (5) measuring plateau pressure at the lower PEEP. The recruited volume is inferred from the excess expiration beyond the predicted tidal volume, allowing calculation of the R/I ratio.

The Recruitment-to-Inflation (R/I) ratio is a recently proposed, dimensionless index that estimates the proportion of tidal volume change attributable to recruitment of previously collapsed alveoli relative to inflation of already open units (54). It is calculated from a single-breath decremental PEEP maneuver, typically involving a drop in PEEP of ≥ 10 cmH₂O (e.g., from 15 to 5 cmH₂O), with measurement of exhaled volume. In order to perform the single breath maneuver, PEEP should be set at 15 cmH2O for at least 10 min, provided that plateau pressure is less than 30 cmH2O and respiratory rate decreased to ensure enough time for complete exhalation. Note the expiratory volume at this PEEP and then change to the lower PEEP value, noting the exhaled volume after the change and the plateau pressure at low PEEP. The recruited volume is inferred from the excess exhalation beyond what is predicted by the compliance at the lower PEEP (Figure 4).

In some patients, particularly with severe ARDS, small airways may remain closed until a critical AOP is reached. If AOP exceeds the initial high PEEP level used in the maneuver, recruitment will be underestimated because certain units remain unopened at baseline. In such cases, increasing the initial PEEP at 5 cmH2O above AOP – if hemodynamically safe – may provide a more accurate R/I ratio.

An R/I ratio of 1.0 indicates that the recruited volume equals the inflation volume of already open lung – reflecting very high recruitability. In contrast, a low R/I ratio suggests that most of the tidal volume change with PEEP adjustment is due to inflation of already aerated regions, implying limited potential benefit from higher PEEP. Importantly, the R/I ratio is a global measure: it reflects the net balance between recruitment and overdistension but cannot localize where these processes occur. Thus, moderate values may mask a combination of regional recruitment and overdistension that cancel each other out. This limitation applies to all gas-based methods, including P–V curve analysis (55). Also, the R/I ratio should be interpreted only within the PEEP range tested during the maneuver.

In clinical practice, the R/I ratio offers a pragmatic, objective means to inform PEEP titration by characterizing lung recruitability at the bedside, potentially improving alignment between physiologic phenotype and ventilatory strategy.

### Prone position

Prone positioning improves respiratory mechanics in ARDS by reducing heterogeneity in transpulmonary pressure. In the supine position, the dependent dorsal lung is compressed by the combined effects of lung weight, mediastinal structures, and abdominal pressure, promoting collapse in posterior regions while favoring relative overdistension of ventral units. Proning patients redistributes these forces, recruits dorsal lung, reduces ventral hyperinflation, and improves ventilation-perfusion matching, improving oxygenation and potentially reducing ventilator-induced lung injury (56, 57).

Prone position ventilation and PEEP are best understood as complementary rather than competing interventions. Both are intended to stabilize lung units, reduce cyclic opening and closing, improve homogeneity of ventilation, and limit injurious stress concentration within the “baby lung.” However, their interaction is complex. Major trials assessing the benefit of prone position in ARDS were conducted mostly with relatively low-PEEP strategies, so the literature does not fully define whether higher PEEP combined with proning is additive or synergistic (58). A post-hoc analysis of these trials showed that most patients received modest post-intervention PEEP, and estimated PEEP would have been substantially higher under high-PEEP protocols, highlighting the limited generalizability of existing trial data to patients managed with aggressive PEEP strategies (58).

Experimental data suggest that PEEP titration strategy modifies the oxygenation response to prone positioning (59). More recent clinical data suggest that this interaction may depend on recruitability: higher PEEP improved V/Q matching in proned patients with high R/I ratio, but worsened V/Q matching in poorly recruitable patients (60).

The patients most likely to benefit from prone positioning are those with moderate-to-severe ARDS, especially severe ARDS with PaO2/FiO2 < 150 mm Hg. The strongest evidence for mortality benefit comes from trials using early proning, prolonged sessions of at least 12 to 16 h per day, and concomitant lung-protective ventilation (61).

### Proposed pragmatic algorithm

**Figure 5:**
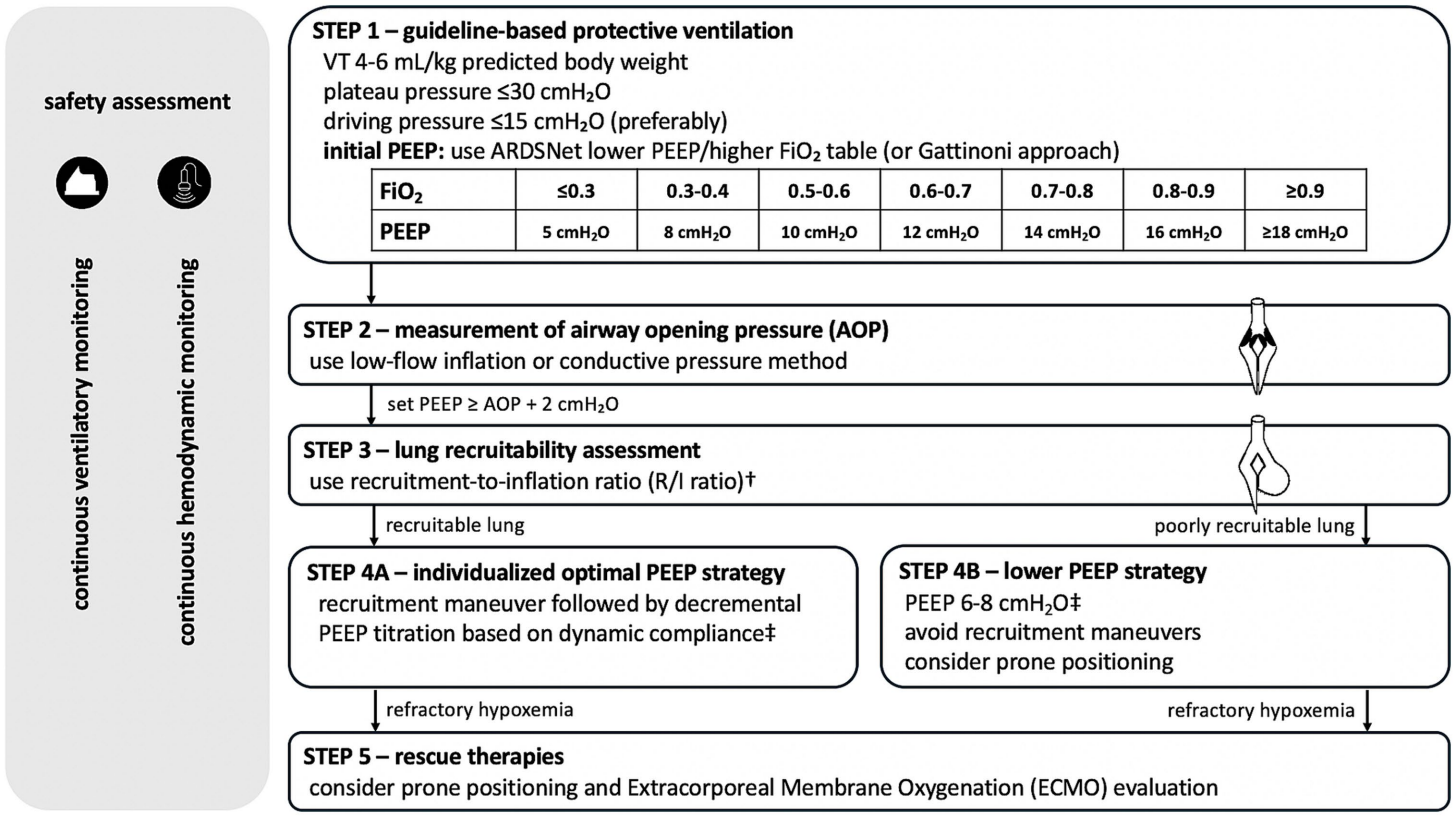
Pragmatic algorithm for PEEP titration in ARDS. Practical bedside algorithm for PEEP (PEEP) titration in patients with acute respiratory distress syndrome (ARDS) receiving invasive mechanical ventilation. † Alternative methods to assess recruitability include computed tomography (CT), pressure–volume (P–V) curve analysis, electrical impedance tomography (EIT), and transpulmonary pressure measurement. ‡ Individualized PEEP titration may also be guided by advanced monitoring techniques such as EIT or transpulmonary pressure.

The following algorithm (Figure 5) provides a pragmatic bedside approach to PEEP titration in patients with ARDS, integrating guideline-based ventilation with individualized physiological assessment using widely available ventilator-derived parameters.

### Step 1–Guideline-based protective ventilation and initial PEEP selection

Following endotracheal intubation, ventilatory settings should adhere to lung-protective principles. VT should be set at 4–6 mL/kg PBW, with plateau maintained ≤30 cmH₂O and driving pressure minimized (preferably ≤15 cmH₂O).

Initial PEEP may be set using a standardized ARDSNet lower PEEP/higher FiO₂ table (62). Alternatively, a pragmatic physiological approach as proposed by Gattinoni et al. (63) may be applied, whereby PEEP is selected according to the severity of hypoxemia (PaO₂/FiO₂ ratio), assuming greater recruitability in more severe ARDS (64). In this framework, lower PEEP levels (approximately 5–10 cmH₂O) are used in milder hypoxemia, while higher PEEP levels (approximately 15–20 cmH₂O) are considered in severe ARDS.

Both approaches provide a practical starting point but do not ensure optimal individualization; therefore, continuous monitoring of respiratory mechanics is essential. Concurrently, continuous monitoring of hemodynamic status, ideally with invasive arterial monitoring and serial echocardiography, should be implemented to detect preload, afterload, or contractility changes that could influence ventilation strategy (65).

### Step 2–Assessment of airway opening pressure

Airway closure may confound interpretation of respiratory mechanics and should be assessed early, using one of the techniques described earlier. When AOP is identified, PEEP should be set at or above this threshold (typically AOP + 2 cmH₂O) to prevent cyclic airway closure and improve the reliability of subsequent measurements.

### Step 3–Lung recruitability assessment

Once airway closure has been addressed, lung recruitability should be evaluated. The R/I ratio provides a simple bedside method based on a single-breath decremental PEEP maneuver. Higher R/I values indicate greater recruitability and help guide subsequent PEEP selection (54). Alternative methods to assess recruitability include computed tomography (CT), pressure–volume (P–V) curve analysis, electrical impedance tomography (EIT), and transpulmonary pressure measurement (66–69).

### Step 4–PEEP strategy according to recruitability

PEEP titration should be individualized according to lung recruitability. In patients with recruitable lungs, typically suggested by a R/I ratio ≥0.5, a higher PEEP strategy may be considered. If there are no contraindications (e.g., significant RV dysfunction, pulmonary cavitation, obstructive lung disease), this may include a cautious stepwise recruitment maneuver followed by a decremental PEEP trial to identify the level associated with the best respiratory system compliance (70). Individualized PEEP titration may also be guided by advanced monitoring techniques such as EIT or transpulmonary pressure (68, 69). In contrast, in poorly recruitable lungs (e.g., R/I ratio <0.5), a lower PEEP strategy is generally preferred, typically in the range of 6–8 cmH₂O or the lowest level compatible with adequate oxygenation and airway patency (≥AOP when present). Recruitment maneuvers should be avoided in this setting.

Irrespective of PEEP strategy, if hypoxemia persists (e.g., PaO₂/FiO₂ < 150), adjunctive therapies such as prone positioning should be considered (60, 61). After prone positioning, subsequent PEEP titration plus further hemodynamic and oxygenation assessment should be performed (58).

### Step 5–Rescue strategies

In patients with refractory hypoxemia despite optimization of ventilatory settings, rescue strategies should be implemented irrespective of lung recruitability. These include prone positioning if not performed previously, and consideration of ECMO in appropriate candidates (71).

## Limitations of the proposed approach

Although the use of ventilation derived parameters to set and individualize PEEP is easily accessible and established in the literature, this approach has many potential disadvantages. First, ventilator derived variables treat the respiratory system as a single homogeneous unit and therefore assessment of lung compliance by this method cannot be separated from chest wall compliance, which may result in incorrect estimation of the stress applied to the lung (72). Targets such as best compliance, lowest driving pressure, or plateau-pressure limits cannot distinguish dependent collapse from nondependent overdistension, and different bedside methods often select markedly different PEEP levels - none of which were associated with recruitability, underscoring that global mechanics alone may misclassify the balance between recruitment and overdistension (66). Protective mechanical ventilation with PEEP application according to the highest compliance has been associated with less organ dysfunction, although no consistent mortality benefit was demonstrated (73).

The use of esophageal pressure monitoring can overcome some of the limitations by separating chest wall from lung compliance, by estimation of the pleural pressure and transpulmonary pressure (72). It has shown in randomized trials improvements in oxygenation/compliance or rescue-therapy use, but no consistent benefit in mortality or ventilator-free days (68, 74).

Similarly, regional assessment of lung collapse and distention is possible through advanced monitoring techniques, such as EIT (75, 76). Furthermore, EIT allows assessment of lung perfusion in addition to lung ventilation and may validate strategies that target a reduction in V/Q mismatch (77). Although it is a promising tool, evidence to date suggests that EIT improves mechanistic endpoints more consistently than patient-centered outcomes (66, 67, 69, 78, 79). Current evidence supports these tools mainly for physiologic personalization rather than as universally proven outcome-improving strategies.

## Conclusion

Positive end-expiratory pressure titration remains a central component of ARDS management, both for preventing VILI and optimizing physiological parameters. Despite decades of physiologic research and multiple clinical trials targeting different interventions, defining the “best” PEEP at the bedside continues to be a complex and unresolved challenge.

Mounting evidence indicates that a uniform approach to PEEP is suboptimal in the context of ARDS heterogeneity. Instead, contemporary strategies emphasize individualized titration, integrating physiologic assessment of recruitability, airway closure, and hemodynamic tolerance.

In this review, we present a pragmatic algorithm grounded in readily available bedside techniques. By systematically addressing airway patency, recruitability, and safety thresholds, this approach aims to balance the competing risks of atelectrauma, overdistension, and hemodynamic compromise. Such physiologically guided strategies may facilitate more precise PEEP titration, potentially improving clinical outcomes while minimizing iatrogenic harm in patients with ARDS.
